# Antennal olfactory responses of adult meadow spittlebug, *Philaenus spumarius*, to volatile organic compounds (VOCs)

**DOI:** 10.1371/journal.pone.0190454

**Published:** 2017-12-29

**Authors:** Giacinto Salvatore Germinara, Sonia Ganassi, Marco O. Pistillo, Carmela Di Domenico, Antonio De Cristofaro, Antonella Marta Di Palma

**Affiliations:** 1 Department of the Sciences of Agriculture, Food and Environment, University of Foggia, Foggia, Italy; 2 Department of Agricultural, Environmental and Food Sciences, University of Molise, Campobasso, Italy; University of Arizona, UNITED STATES

## Abstract

The meadow spittlebug, *Philaenus spumarius* L. (Hemiptera, Aphrophoridae) is a commonly found vector of *Xylella fastidiosa* Wells et al. (1987) strain subspecies *pauca* associated with the “Olive Quick Decline Syndrome” in Italy. To contribute to the knowledge of the adult *P*. *spumarius* chemoreceptivity, electroantennographic (EAG) responses of both sexes to 50 volatile organic compounds (VOCs) including aliphatic aldehydes, alcohols, esters, and ketones, terpenoids, and aromatics were recorded. Measurable EAG responses were elicited by all compounds tested. In both sexes, octanal, 2-octanol, 2-decanone, (*E*)-2-hexenyl acetate, and vanillin elicited the strongest antennal amplitude within the chemical groups of aliphatic saturated aldehydes, aliphatic alcohols, aliphatic acetates and aromatics, respectively. Male and female EAG responses to sulcatol, (±)linalool, and sulcatone were higher than those to other terpenoinds. In both sexes, the weakest antennal stimulants were phenethyl alcohol and 2-pentanone. Sexual differences in the EAG amplitude were found only for four of test compounds suggesting a general similarity between males and females in antennal sensitivity. The olfactory system of both sexes proved to be sensitive to changes in stimulus concentration, carbon chain length, and compound structure. Compounds with short carbon chain length (C_5_—C_6_) elicited lower EAG amplitudes than compounds with higher carbon chain length (C_9_—C_10_) in all classes of aliphatic hydrocarbons with different functional groups. The elucidation of the sensitivity profile of *P*. *spumarius* to a variety of VOCs provides a basis for future identification of behaviorally-active compounds useful for developing semiochemical-based control strategies of this pest.

## Introduction

The meadow spittlebug, *Philaenus spumarius* L. (Hemiptera: Aphrophoridae) is a species widespread in the Holarctic Region [1]. Hundreds of *P*. *spumarius* host plants have been recorded ranging from grasses to trees, including meadow crops, herbs, garden plants and it was observed that dicotyledonous plants tend to be used more often than monocotyledonous ones [2, 3]. In particular, nitrogen fixing herbaceous legumes and some other plants which have a high amino acid concentration in the xylem sap (*Medicago sativa* L., *Trifolium* spp., *Vicia* spp.) are most favored [4]. The insect is univoltine with overwintering eggs [5, 6]. Nymphs and adults are “xylem-feeders” on nearly all parts of the plants above soil level, but mainly on actively growing parts [7, 8]. They damage plants both directly by sucking great amounts of sap which results in plant weakening, deformations and reduced yield [9], and indirectly by vectoring some important plant diseases including the grapevine Pierce’s disease [10], the peach yellows and little peach disease [7].

In Europe, *P*. *spumarius* rarely caused severe damage and it has not been considered a serious pest for several years [11, 12]. However, it was recently shown to be a commonly found vector of *Xylella fastidiosa* Wells et al. (1987) strain subspecies *pauca* associated with the “Olive Quick Decline Syndrome” in the Salento Peninsula (Southern, Italy) due to the insect’s capability of acquiring and inoculating the bacterium from/to different host plants [13, 14, 15]. Furthermore, because *X*. *fastidiosa* is persistent in insect vectors including spittlebugs [16], *P*. *spumarius* adults may inoculate olive trees over an extended period of time. The meadow spittlebug was the most abundant species found in Southern Apulia orchards on both weeds and olive trees and the *X*. *fastidiosa* prevalence in *P*. *spumarius* on olive trees was approximately twice than that in insects collected from weeds [15]. These observations drastically changed the pest status of *P*. *spumarius* in Europe where it is currently regarded as a very serious pest requiring the urgent implementation of effective control measures. To this end, the identification of *P*. *spumarius* semiochemicals to manipulate the insect behavior could contribute to the development of new and sustainable control means. For example, phytophagous insects rely on allelochemicals (*sensu* Nordlund and Lewis) [17] in the search for food, mate, and egg-laying sites and to avoid suboptimal substrates [18, 19]. Allelochemicals able to attract insects (kairomones) can be used to develop suitable pest monitoring tools whereas compounds able to repel insects (allomones) have the potential to provide direct control through deterring pests from food and oviposition sites [19, 20, 21].

Electroantennography (EAG) is used to identify candidate behaviourally-active compounds. An EAG profile represents the sensitivity of olfactory receptor neurons on the antennae that are tuned to chemicals tested and EAG-active compounds are frequently of ecological significance [22]. *P*. *spumarius* was shown to possess a low number of antennal sensory structures if compared to other leafhopper and planthopper species but the general organization of some basiconic and coeloconic sensilla is consistent with an olfactory function [23].

The present study was designed to assess the capability of the peripheral olfactory system of male and female *P*. *spumarius* adults to perceive plant volatile organic compounds (VOCs) using the EAG technique. The EAG responses of male and female insects were also compared.

## Materials and methods

### Insects

Nymphs of *P*. *spumarius* were collected from *M*. *sativa* plants in privately owned lands near the University of Foggia (41°27' N, 15°30' W) (Apulia Region, Italy). Permission to collect insect samples was obtained from the owners. Nymphs were transferred to plexiglas cages on shoots of *Vicia faba* L. seedlings maintained at 23±2°C, 70±5% relative humidity (r.h.), and 14:10 L:D photoperiod. Emerged adults were collected daily and provided with fresh *V*. *faba* seedlings in adult cages. For microscopic observations and EAG recordings one to two-day-old males and females were used. The sex of the insects tested was determined by observing their genitalia with a stereomicroscope. Before the EAG experiments, insects were kept individually in glass vials (1.5 cm diameter x 5 cm) in the absence of plant odors for at least 4 h.

### Scanning electron microscopy (SEM)

In order to get reliable EAG recordings, the precise location of the sensilla on adult *P*. *spumarius* antennae was investigated by scanning electron microscope (SEM). Males and females of *P*. *spumarius* were stored in 70% ethanol. After dehydration through a graded ethanol series, they were dried using a Baltec CPD030 critical point dryer. Hence, using a stereomicroscope, the antennae were removed and mounted with different orientations on SEM stubs using conductive carbon adhesive tabs and sputter coated with palladium using a Baltec SCD005 coating apparatus. Specimens were observed and photographed with a Hitachi TM3030 tabletop microscope.

### Odor stimuli

Test compounds were 50 VOCs selected to represent different chemical classes including aliphatic alcohols, aldehydes, esters, and ketones, terpenoids, and aromatics (Table 1).

**Table 1:** EAG responses of *P*. *spumarius* adults to a range of volatile organic compounds (VOCs).

Class Compound^a^	Chemical purity (%)	Absolute EAG response in mV (Mean ± SE)	
		Males	Females
*Aliphatic aldehydes*			
Pentanal	95.0	0.22 ± 0.03	0.22 ± 0.03
Hexanal	98.0	0.28 ± 0.04	0.33 ± 0.07
Heptanal	95.0	0.40 ± 0.05	0.32 ± 0.06
Octanal	99.0	0.52 ± 0.04	0.43 ± 0.05
Nonanal	95.0	0.43 ± 0.05	0.41 ± 0.06
Decanal	95.0	0.33 ± 0.03	0.30 ± 0.04
(*E*)-2-Hexenal	99.0	0.26 ± 0.03	0.26 ± 0.04
(*E*)-2-Heptenal	97.0	0.35 ± 0.03	0.32 ± 0.03
(*E*)-2-Octenal	94.0	0.43 ± 0.05	0.37 ± 0.03
(*E*)-2-Nonenal	97.0	0.48 ± 0.08	0.43 ± 0.05
(*E*)-2-Decenal	95.0	0.53 ± 0.08	0.41 ± 0.06
(*E*,*E*)-2,4-Hexadienal	95.0	0.30 ± 0.03	0.22 ± 0.04
(*E*,*E*)-2,4-Heptadienal	88.0	0.24 ± 0.03	0.24 ± 0.04
(*E*,*E*)-2,4-Nonadienal	85.0	0.20 ± 0.02	0.20 ± 0.02
(*E*,*E*)-2,4-Decadienal	85.0	0.20 ± 0.02	0.22 ± 0.03
*Aliphatic alcohols*			
1-Pentanol	99.0	0.28 ± 0.06	0.29 ± 0.04
3-Pentanol	98.0	0.13 ± 0.03	0.11 ± 0.02
1-Hexanol	98.0	0.16 ± 0.03	0.13 ± 0.01
1-Heptanol	98.0	0.39 ± 0.05	0.29 ± 0.03
1-Octanol *	98.0	0.32 ± 0.05	0.20 ± 0.02
2-Octanol	96.0	0.46 ± 0.06	0.39 ± 0.06
1-Nonanol	98.0	0.23 ± 0.03	0.18 ± 0.03
1-Decanol	98.0	0.21 ± 0.04	0.20 ± 0.02
3-Methyl-1-butanol	99.0	0.26 ± 0.05	0.35 ± 0.04
*Aliphatic ketones*			
2-Pentanone	97.0	0.08 ± 0.02	0.09± 0.02
2-Hexanone	99.0	0.13 ± 0.01	0.18 ± 0.02
2-Heptanone	98.0	0.13 ± 0.02	0.11 ± 0.01
2-Octanone	98.0	0.24 ± 0.03	0.25 ± 0.02
2-Nonanone	99.0	0.19 ± 0.02	0.24 ± 0.03
2-Decanone	98.0	0.27 ± 0.04	0.30 ± 0.03
2-Undecanone	99.0	0.14 ± 0.03	0.16 ± 0.02
2-Tridecanone	99.0	0.19 ± 0.02	0.20 ± 0.02
*Aliphatic esters*			
(*Z*)-3-Hexenyl acetate	98.0	0.20 ± 0.02	0.23 ± 0.03
(*E*)-2-Hexenyl acetate	98.0	0.24 ± 0.03	0.24 ± 0.02
*Terpenes*			
α-Pinene	98.0	0.10 ± 0.02	0.15 ± 0.03
β-Pinene	98.0	0.12 ± 0.02	0.16 ± 0.02
Limonene	97.0	0.26 ± 0.04	0.22 ± 0.03
α-Farnesene	95.0	0.22 ± 0.02	0.20 ± 0.02
β-Caryophyllene	80.0	0.14 ± 0.02	0.21 ± 0.03
Myrcene	92.0	0.21 ± 0.03	0.28 ± 0.03
1.8-Cineole *	99.0	0.16 ± 0.01	0.24 ± 0.03
(±)Linalool *	99.0	0.33 ± 0.03	0.48 ± 0.05
Sulcatol (6-Methyl-5-hepten-2-ol)	99.0	0.37 ± 0.05	0.49 ± 0.07
Sulcatone (6-Methyl-5-hepten-2-one)	99.0	0.39 ± 0.03	0.46 ± 0.07
*Aromatics*			
Phenylacetaldehyde **	90.0	0.11 ± 0.01	0.19 ± 0.02
Methylsalicylate	99.0	0.19 ± 0.02	0.19 ± 0.02
Benzaldehyde	99.0	0.16 ± 0.01	0.14 ± 0.04
Vanillin	99.0	0.27 ± 0.02	0.28 ± 0.04
Phenethyl alcohol	99.0	0.03 ± 0.01	0.06 ± 0.02

^a^ Asterisks indicate significant differences between sexes (* P = 0.05, ** P = 0.01, *t*-test).

In order to prevent rapid evaporation of test compounds, they were dissolved in mineral oil (Sigma-Aldrich, Milan, Italy). For each compound, a 10 μg/μL solution was prepared. To obtain dose-response curves, mineral oil solutions (0.001, 0.01, 0.1, 1, 10, 100 μg/μL) of (*Z*)-3-hexenol, used as a standard compound, were also prepared. Solutions were stored at -20°C until needed. Just before the experiment, 10 μL of each test solution was adsorbed onto a filter paper strip (1 cm^2^, Whatman No. 1) placed in a Pasteur pipette (15 cm long), which served as an odor cartridge.

### EAG recordings

The EAG technique was similar to that used in previous studies [24, 25]. The head of the insect was dissected and the distal half of the arista removed. A glass pipette filled with Kaissling saline [26] that served as the indifferent electrode was inserted into the base of the head. The tip of the amputated arista was put in contact with the end of a similar pipette (0.1 mm diameter) which provided the recording electrode.

**Fig 1:**
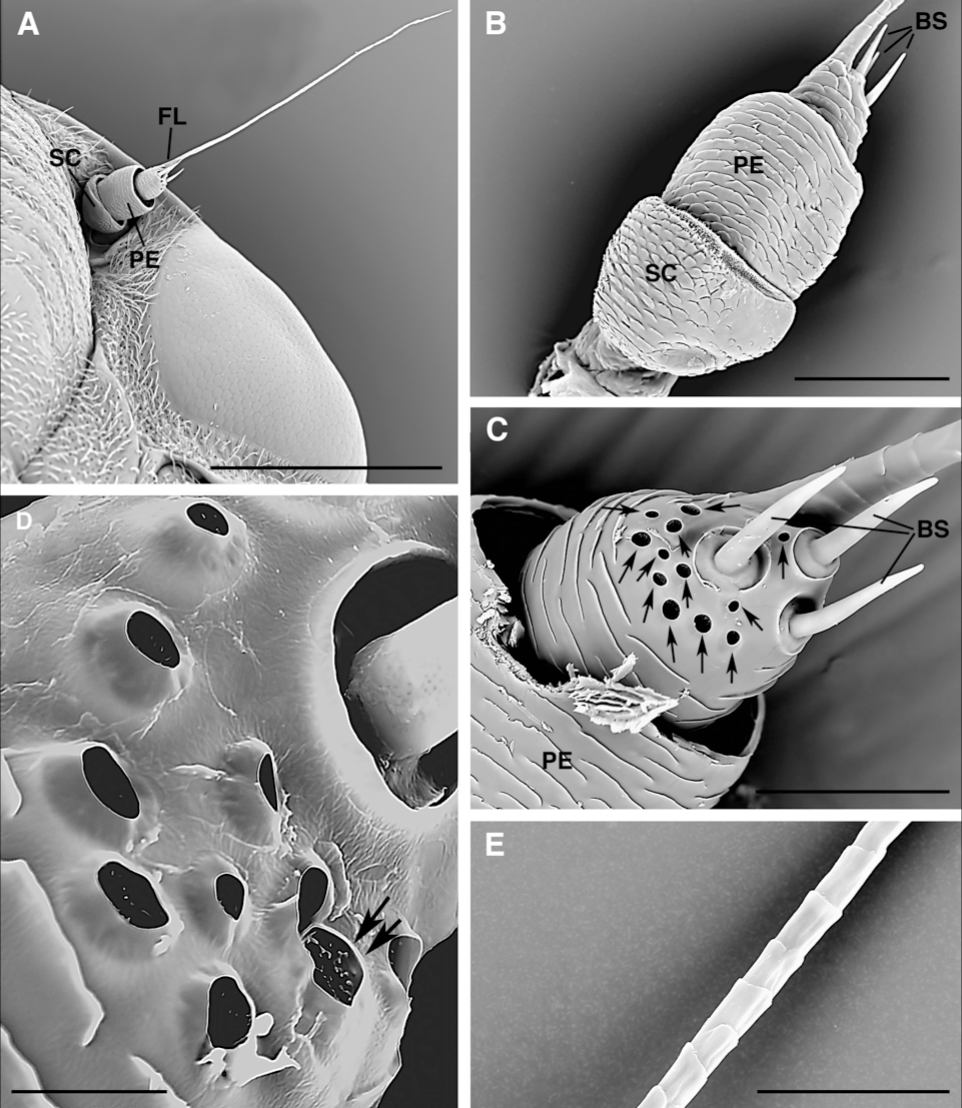
*Philaenus spumarius* SEM: Antenna. A) Overview of the antenna composed by three segments: scape, pedicel and the long flagellum. B) Closer dorsal view of the scape, pedicel and proximal larger region of the flagellum bearing the three basiconic sensilla. C) Enlargement of the basal region of the flagellum showing the basiconic (BS) and coeloconicum sensilla (arrows) located on the side facing the compound eyes (adaxial surface) when the antenna is inserted on the head. D) Detail of the coeloconicum sensilla showing the microtrichia guarding the opening (arrows) of the pits. E) Detail of the regular cuticular scales covering the elongated thread-like part of the flagellum. Abbr.: BS, basiconic sensillum; SC, scape; PE, pedicel; Fl, flagellum; Scale bar: 500 μm (A); 100 μm (B); 50 μm (C); 10 μm (D); 30 μm (E).

AgCl-coated silver wires were used to maintain the electrical continuity between the antennal preparation and an AC/DC UN-6 amplifier in DC mode connected to a PC equipped with the EAG 2.0 program (Syntech Laboratories, Hilversum, The Netherlands). Stimuli were blown by a disposable syringe into a constant stream of charcoal-filtered humidified air (500 mL/min) flowing in a stainless steel delivery tube (1 cm diameter) with the outlet positioned at approximately 1 cm from the antenna. Based on SEM observations, the adaxial surface of the antenna housing basiconic and coeloconic sensilla (Fig 1A, 1C and 1D) with a putative olfactory function was exposed to the air flow. Over 1 s, 2.5 cm^3^ of vapor from an odor cartridge were added. In dose-response experiments, stimuli were applied in ascending dose [27] whereas in the other experiments they were randomly sequenced. Control (10 μL of mineral oil) and standard (10 μL of a 10 μg/μL (Z)-3-hexenol solution) stimuli were applied at the beginning of the experiment and after each group of 7 test odors. Intervals between stimuli were 30 s. For each compound, EAG responses were recorded from 8 antennae of different insects of each sex.

### Data analysis

EAG responses were evaluated by measuring the maximum amplitude of negative polarity deflection (-mV) elicited by a stimulus [28]. The absolute value of the EAG amplitude (mV) to each test stimulus was adjusted to compensate for solvent and/or mechanosensory artifacts by subtracting the mean EAG response of the two nearest mineral oil controls [29]. The resulting EAG amplitude was corrected according to the reduction of the EAG response to the standard stimulus to compensate for the decrease of the antennal responsiveness during the experiment [30]. No corrections were made for differences in volatility between the test compounds. Consequently, comparisons among responses are relative.

Male and female mean EAG responses to each compound were compared using independent samples Student’s *t*-test (P = 0.05). In dose-response curves, the activation threshold was considered to be the first dose at which the mean response was higher than “0” value using Shapiro-Wilk test for normality followed by one-sample Student’s *t*-test (P = 0.05); saturation level was taken as the lowest dose at which the mean response was equal to or less than the previous dose [31].

To evaluate antennal activation, within each sex, the corrected EAG responses to each compound were compared to “0” value using Wilcoxon rank sum test and regarded as “measurable” if significant at P = 0.05. Analyses were performed with SPSS (Statistical Package for the Social Sciences) version 10.0.7 for Windows (SPSS Inc., Chicago, IL).

## Results

### Localization of antennal sensilla

The antenna of *P*. *spumarius*, both in male and female, is rather small and composed of three segments (Fig 1A). The first two, the scape and the pedicel, are short almost cylindrical, covered by cuticular scales (Fig 1A and 1B). The third segment, the flagellum, presents a proximal larger part, still short, that carries 3 peg-like basiconic sensilla (Fig 1B and 1C) and 12 coeloconic sensilla (i.e. pegs in pits) (Fig 1C and 1D) while the rest of the flagellum is represented by a very elongated thread-like part (Fig 1A), covered by regular cuticular scales (Fig 1E). The coeloconic sensilla are grouped on the adaxial surface of the antennal segment i.e. the side facing the compound eyes (Fig 1A and 1C) and in semicircle around the basiconic sensilla (Fig 1C).

### Antennal sensitivity

**Fig 2:**
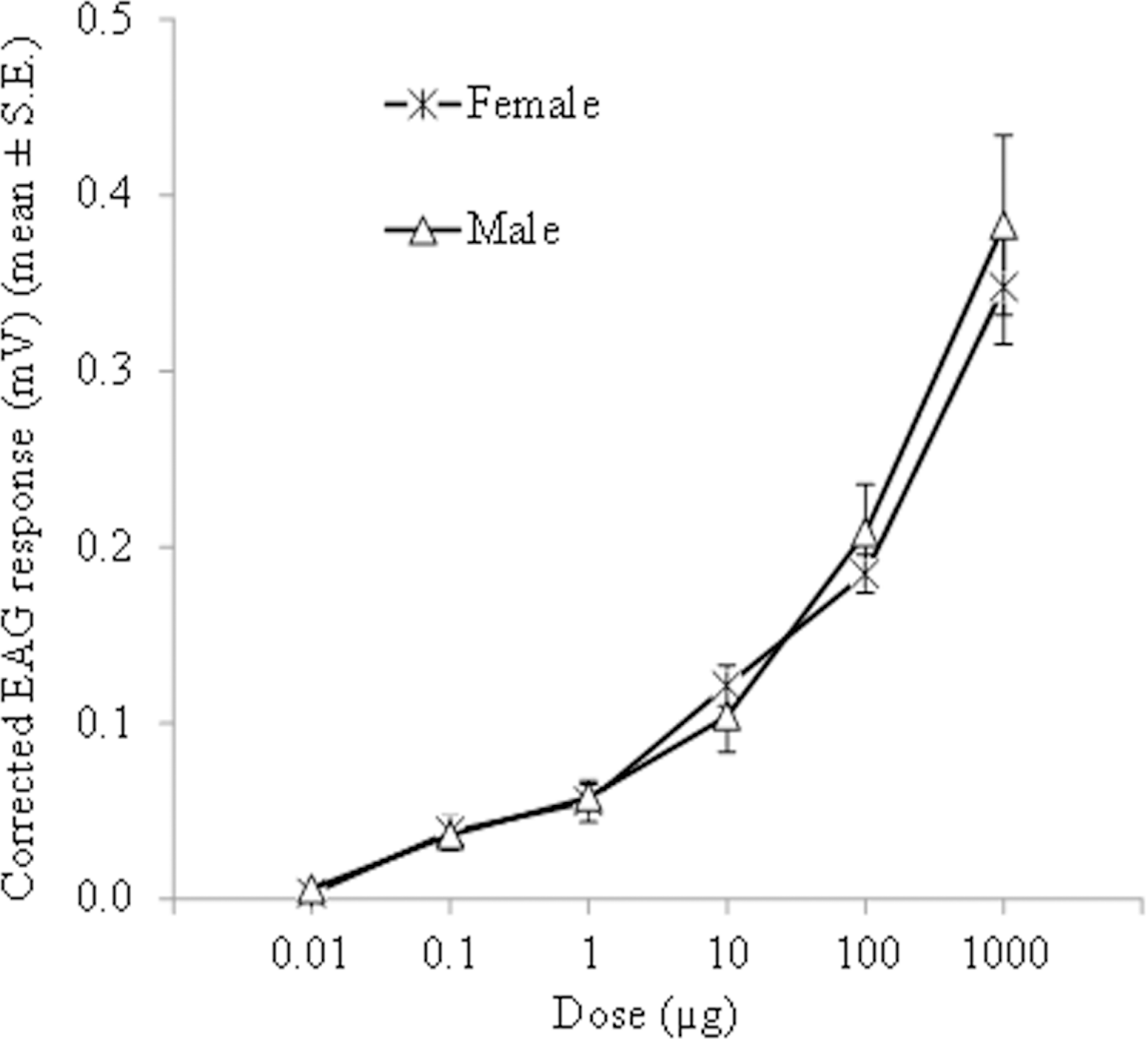
EAG dose-response curves of female and male antennae of *P*. *spumarius* adults to ascending doses of (*Z*)-3-hexen-1-ol.

The sensitivity of adult *P*. *spumarius* antennae towards increasing doses of (*Z*)-3-hexenol is reported in Fig 2. In the dose range tested, the mean EAG responses varied from 0.003 ± 0.003 to 0.348 ± 0.032 mV for females and from 0.006 ± 0.003 to 0.383 ± 0.051 mV for males (S1 Table). The activation threshold was 0.1 μg for both sexes (P < 0.05; one-sample *t*-test). Male and female EAG responses increased from 100 to 1000 μg indicating no saturation of antennal receptors at the lower dose. At all doses tested, the mean EAG responses were not significantly different (*t* = 0.082–1.341; d.f. 14; P ≥ 0.05) between males and females.

### Antennal selectivity

The mean EAG responses of males and females to test stimuli are shown in Table 1. All compounds tested elicited measurable EAG responses in both sexes (P < 0.05 in all Wilcoxon rank sum test). Responses ranged from 0.06 ± 0.02 mV (phenethyl alcohol) to 0.49 ± 0.07 mV (6-methyl-5-hepten-2-ol) in females (S2 Table) and from 0.03 ± 0.01 mV (phenethyl alcohol) to 0.53 ± 0.8 mV [(*E*)-2-decenal] in males (S3 Table).

Among all compounds tested, the largest EAG amplitudes (> 0.4 mV) were observed in response to *(E)*-2-decenal, octanal, (*E*)-nonenal, 2-octanol, (*E*)-2-octenal, nonanal, heptanal, in males, and to 6-methyl-5-hepten-2-ol, linalool, 6-methyl-5-hepten-2-one, (*E*)-2-nonenal, octanal, *(E)*-2-decenal, nonanal in females. In both sexes, the weakest antennal stimulants (< 0.10 mV) were phenethyl alcohol and 2-pentanone. For four compounds, significant differences between females and males in the magnitude of their EAG responses were found. Males showed a significantly higher EAG response than females to 1-octanol (*t* = 2.32, d.f. 14, P < 0.05) whilst female responses significantly exceeded those of males for 1,8-cineole (*t* = 2.96, d.f. 14, P < 0.05), linalool (*t* = 2.83, d.f. 14, P < 0.05) and phenylacetaldehyde (*t* = 4.65, d.f. 14, P < 0.01) (Table 1).

**Fig 3:**
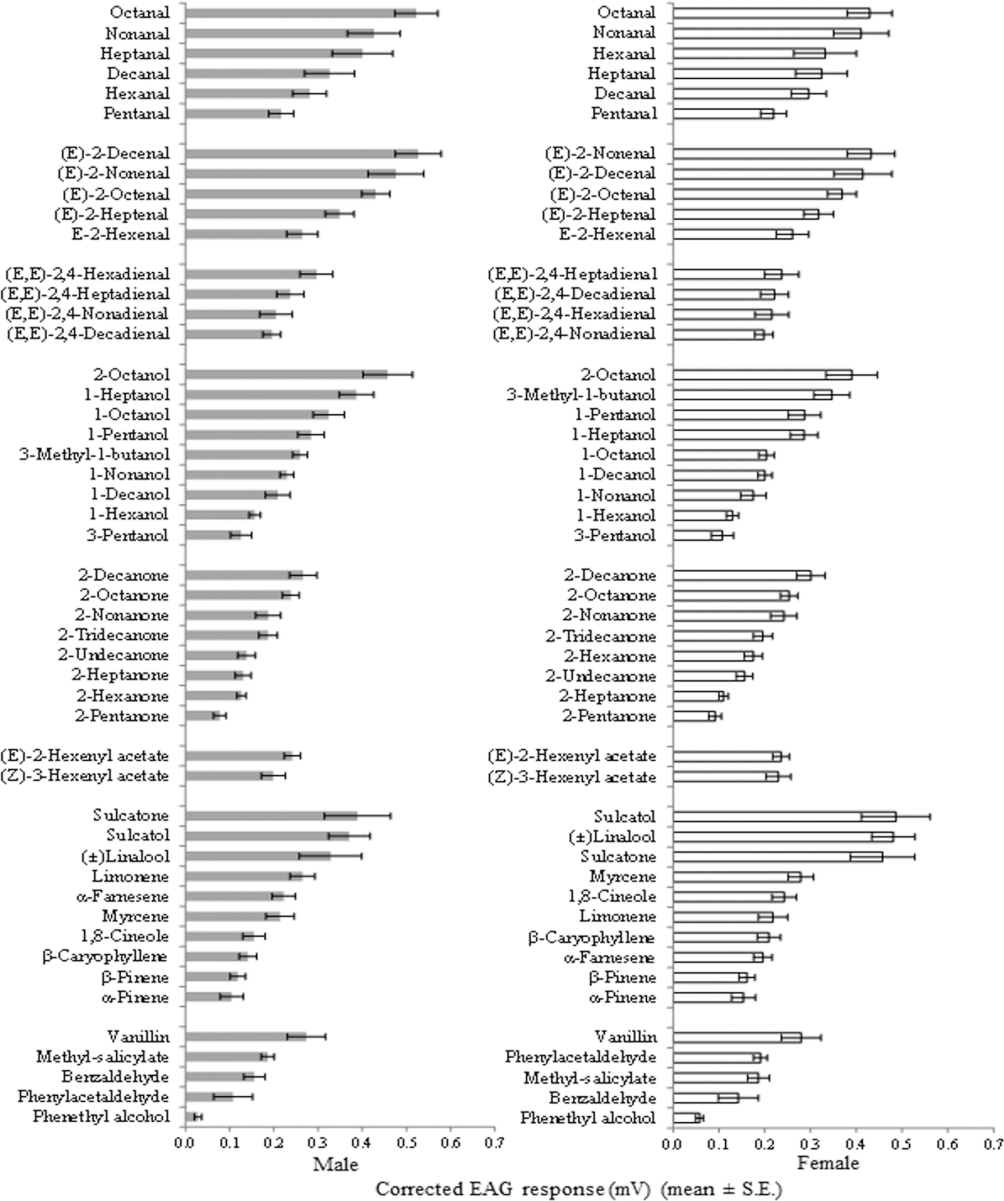
EAG response profile of adult *P*. *spumarius* to a range of VOCs. Within each chemical group, compounds are listed according to the mean amplitude of the EAG response (n = 8) elicited at the 100 μg dose.

In both sexes, octanal, 2-octanol, 2-decanone, (*E*)-2-hexenyl acetate, and vanillin elicited the strongest antennal amplitude within the chemical groups of aliphatic saturated aldehydes, aliphatic alcohols, aliphatic acetates and aromatics, respectively (Fig 3). Within aliphatic monounsaturated aldehydes, the highest EAG responses were induced by (*E*)-2-decenal in males and (*E*)-2-nonenal in females. In both sexes, mean EAG responses to sulcatol, (±)linalool, and sulcatone were higher than those to other terpenoids.

Considering antennal responsiveness of both sexes to aliphatic hydrocarbons with different functional groups, compounds with the shortest carbon chain length (C_5_—C_6_) elicited lower EAG amplitudes than compounds with higher carbon chain length (C_9_—C_10_) in all chemical classes. This was particularly evident for the aliphatic monounsaturated aldehydes and ketones. Antennal sensitivity to C_7_, C_9_, and C_10_ diunsaturated compounds was lower than that to the corresponding saturated and/or monounsaturated analogs (Fig 3).

## Discussion

EAG preparations set up by exposing the adaxial surface of adult *P*. *spumarius* flagellum to the air stream flowing on the antenna enabled us to get reliable EAG recordings on stimulation with different VOCs.

Since an EAG response represents the summation of generator potentials of simultaneously stimulated receptor cells by an odor stimulus [32, 33, 34], results of this study confirm the olfactory function of the basiconic and the 8 double-walled coeloconic sensilla present on the spittlebug flagellum as previously hypothesized [23] according to their external and internal organization (i.e. porous sensillar wall and highly branched dendrites). Moreover, in spite of the low number of antennal sensory structures, all test compounds elicited measurable EAG responses in both sexes, thus demonstrating the capability of the peripheral olfactory systems to perceive a broad range of VOCs.

Among spittlebugs, antennal sensitivity to VOCs has been demonstrated for the rice spittlebug, *Callitettix versicolor* (Fabricius) (Hemiptera, Cercopidae), whose nymphs displayed EAG responses to six *n*-alkanes (C_11_—C_16_) which were characterized as components of a self-regulatory pheromone system that controls nymph aggregation behavior [35].

Among the 50 compounds tested, only four compounds elicited significantly different EAG responses between males and females suggesting a general similarity between sexes in antennal sensitivity. This is in concurrence with the similarity between sexes in the number and distribution of antennal sensilla and suggests common ecological needs, i.e. for host habitat and/or host plant selection. A general similarity between male and female EAG responses to plant volatiles has been reported for many other insect species including *Leptinotarsa decemlineata* (Say) [36], *Yponomeuta* species and *Adoxophyes orana* (Fisch. v. Roesl.) [37], and some tephritid fruit flies [22, 38, 39].

The antennal responsiveness profiles to systematically assessed carbon-chain series of three functional-group classes and comparison of saturated, mono- and diunsaturated aliphatic aldehydes showed that in both sexes the amplitude of EAG responses did not correlate with the molecular weight and, therefore, the volatility of compounds tested. Overall, among aliphatic alcohols, aldehydes, and ketones, male and female EAG responses to C_9_ and C_10_ compounds were higher than those elicited by more volatile C_5_ and C_6_ compounds. Both sexes were more responsive to C_9_ and C_10_ saturated and monounsaturated aldehydes than more volatile diunsaturated analogs. These observations confirm that differences in the EAG amplitudes mainly depend on the sensitivity of the olfactory system of the species under study [37, 40], even though differences in volatility of test compounds may result in different numbers of molecules reaching the EAG preparation [41]. Consequently, the high sensitivity of the olfactory system to specific compounds suggests a their possible ecological relevance. EAG experiments also showed that male and female spittlebug antennae are sensitive to changes in stimulus concentration. In fact, dose-dependent EAG responses were found in both sexes on stimulation with increasing concentrations of (*Z*)-3-hexenol.

Over the range of VOCs tested, the strongest antennal simulants were (*E*)-2-decenal followed by octanal and (*E*)-nonenal in males and 6-methyl-5-hepten-2-ol followed by linalool and 6-methyl-5-hepten-2-one in females. All these compounds have been previously identified among VOCs emitted by different plant and insect species and they are known to play different infochemical functions in the chemical communication of many insect species [42]. For instance, (*E*)-2-decenal has been identified from several plants including *Olea europaea* L. (Oleaceae) [43, 44], in the scent of *Podisus* and *Supputius* species (Heteroptera, Pentatomidae) [45] and the predatory bug *Geocoris punctipes* (Say) (Hemiptera, Lygaeidae) [46] and it can act as a pheromone, an attractant or an allomone depending on the insect species. The compound 6-methyl-5-hepten-2-ol (sulcatol) was found in the floral scent of many plant species, in the blend of volatiles emitted by wheat seedlings heavily infested by bird cherry-oat aphid, *Rhopalosiphum padi* (L.) (Homoptera, Aphididae), and it is also synthetized in the hindgut of males of the ambrosia beetle *Megaplatypus mutatus* (Chapuis) (Coleoptera, Platypodidae) which release it to attract females [47]. This compound, in a naturally occurring mixture with 6-methyl-5-hepten-2-one and 2-tridecanone, mediates spacing behavior of *R*. *padi* feeding on cereals [48] and is the aggregation pheromone of several ambrosia beetles [49, 50, 51].

In conclusion, this study provides evidence on the functionality of antennal sensilla previously described as putative olfactory sensilla in *P*. *spumarius* adults. Moreover, it demonstrates the capability of the male and female olfactory systems to selectively perceive a variety of VOCs with a possible infochemical role that, alone or in combination with visual and vibrational stimuli, may modulate *P*. *spumarius* intra- and interspecific interactions. This first contribution to the knowledge of the sensitivity profile of *P*. *spumarius* to a variety of VOCs provides a basis for future chemical, electrophysiological and behavioral investigations aimed at identifying biologically-active compounds useful for the implementation of semiochemical-based control strategies for this pest.

## Supporting information

S1 Table Corrected EAG responses of *Philaenus spumarius* females (n = 8) and males (n = 8) to increasing doses of Z3-hexenol. (XLSX)

S2 Table Corrected EAG responses of individual *Philaenus spumarius* females (n = 8) to a range of volatile organic compounds (VOCs) and to Z3-hexenol and mineral oil respectively used as standard and control stimuli during each experiment. (XLSX)

S3 Table Corrected EAG responses of individual *Philaenus spumarius* males (n = 8) to a range of volatile organic compounds (VOCs) and to Z3-hexenol and mineral oil respectively used as standard and control stimuli during each experiment. (XLSX)
